# The Development of the Food Averse Questionnaire: A Measure of Food Avoidance in Children With and Without Autistic Spectrum Conditions

**DOI:** 10.1111/mcn.70025

**Published:** 2025-04-22

**Authors:** Maria Pomoni, Gillian Harris, Helen Coulthard

**Affiliations:** ^1^ School of Psychology University of Birmingham, Edgbaston Birmingham UK; ^2^ Education Endowment Foundation London UK; ^3^ School of Applied Social Sciences De Montfort University Leicester

**Keywords:** autism, avoidant eating, child eating, fussy, neophobia, picky, sensory

## Abstract

The aim of this study was to 1) develop a measure of avoidant eating behaviours for both typically developing children (TD), and those with Autism Spectrum Conditions (ASC), and 2) to examine whether these current behaviours are associated with reports of early feeding difficulties in both populations. In study one (*n* = 336) parents of 4‐ to 14‐year‐old children completed a series of questions about food avoidance. Three subscales of food avoidance were identified with a total scale of 31 items; avoidant, rigid‐inflexible, and texture sensitive. Analyses found that scores on these subscales were associated with related measures of picky eating, food neophobia, sensory sensitivity and cognitive inflexibility, as well as lower fruit, vegetable, dairy and protein consumption. In study two, 225 children aged 4–14 years and their parents were recruited (143 TD and 78 ASC). Children with ASC were more likely to have feeding problems during the transition to family foods and in the toddler eating period in comparison to TD children. Additionally, children with ASC showed, at the time of the study, higher avoidance, rigid‐inflexible eating and texture‐sensitive eating behaviours than TD children. This study has developed a reliable scale for food avoidance for children with and without ASC diagnoses. Food avoidance is more severe in children with ASC than in TD children and these difficulties may start before them receiving an ASC diagnosis. Further work is needed to examine the usefulness of this scale in clinical and nonclinical populations.

## Summary

- The Food AVERSE questionnaire is a novel scale to measure avoidant eating behaviours in children with and without ASC.
- The Food AVERSE questionnaire comprises three factors; food avoidance, rigid‐inflexible and texture sensitive.
- Children with ASC had higher scores on the Food AVERSE Questionnaire and were also reported to have more problems with feeding before their diagnosis.
- This scale can be used in clinical and nonclinical samples of children to measure different aspects of food avoidance.

## Graphical abstract

The Food AVERSE questionnaire measures avoidant eating in children aged 4–14 years. It has three subscales which measure food avoidance, rigid‐inflexible eating and texture‐sensitive eating. The scale is a useful measure to look at avoidant eating across different children and has been tested on both typically developing and autistic children.

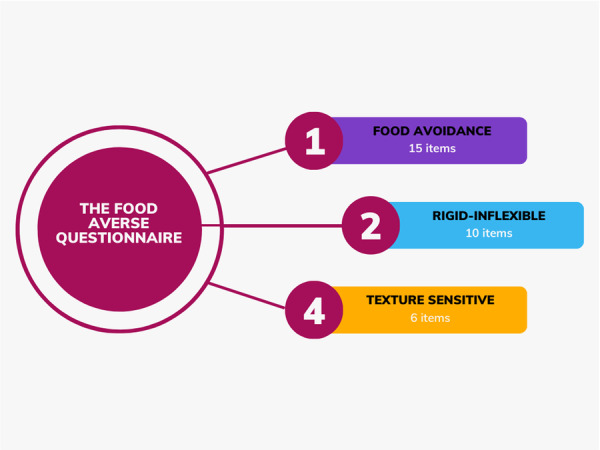

## 1 Introduction

Avoidant eating, also known as picky/fussy/selective eating, refers to the rejection or the lack of interest in a substantial number of familiar and novel foods (Lafraire, et al. 2016; Taylor and Emmett 2019). Avoidant eaters follow a diet which is more likely to be lower in nutrients such as zinc and iron (Saati and Adly 2023; Taylor et al. 2016; Drayton et al. 2023) and have a persistent pattern of dietary intake lower in fruits, vegetables, and meats than non‐avoidant children (Taylor and Emmett 2019). Avoidant eaters will have strong preferences in the appearance or presentation of their food (Brown and Harris 2012; Thomas et al. 2021) and some may refuse specific textures (Coulthard and Sahota 2016; Coulthard et al. 2022). Avoidant eating behaviour is a feature of the clinical diagnosis of avoidant restrictive food intake disorder (ARFID) in the Diagnostic and Statistical Manual (DSM) 5 (American Psychiatric Association 2013), where extreme food avoidance and restriction leads to deficits in physiological, nutritional and/or psychosocial functioning (Bourne et al. 2020; Silvers and Erlich 2023).

Avoidant eating is a common behaviour in some children diagnosed with autistic spectrum conditions (ASC) and is among the major reasons for the referral of children with ASC to nutritional services (Nadeau et al. 2022). Previous literature has also indicated a high prevalence of children with ASC who are underweight or show extreme food avoidance, eating only 5–6 or fewer different foods (Kahathuduwa et al. 2022; Keen 2008). There are a number of factors that could underpin this avoidance. Individuals with ASC have also been shown to be more likely to insist on the same foods or have ritualistic behaviour during mealtimes (Bourne et al. 2022; Esteban‐Figuerola et al. 2019). Sensory sensitivity is more prevalent in children with ASC (Tonacci et al. 2017) as a result, the sensory components of food such as texture, taste and smell are also more likely to affect the food choices of children with ASC (Zulkifli et al. 2022).

Increased interest in normal and clinical manifestations of avoidant eating needs reliable and valid measures upon which to base research studies. Traditional measures of avoidant/picky/fussy eating refer to behaviours that encompass both familiar and novel food rejection (Lafraire et al. 2016). The most commonly used measure is the food fussiness subscale of the Child Eating Behaviour Questionnaire (CEBQ; Wardle et al. 2001), which mainly measures food neophobia (four of the six items) and the Child Food Rejection Scale (CFRS; Rioux et al. 2017) which separates out food neophobia and familiar food rejection into separate subscales. Recent research has factored the eight subscales of the CEBQ into food approach and food avoidance measures (Vandeweghe et al. 2016). The food avoidance measure sums the subscales of food fussiness, satiety responsiveness, slowness in eating and emotional undereating. Whilst combined, these 19 items reflect low appetite and food neophobia well, but they do not reflect the full range of avoidant eating behaviours seen in nonclinical and clinical samples, such as texture sensitivity or mealtime rituals (Wolstenholme et al. 2022; Taylor and Emmett 2019).

Several scales have been used to examine a range of eating behaviours in children with ASC and in clinical populations. The Brief Autism Mealtime Behaviour Inventory (BAMBI; Lukens and Linscheid 2008) and a more recent domain‐specific scale called the Aut‐Eat (Gal et al. 2022) are measures of eating problems in autistic children. There is also the Behavioural Paediatrics Feeding Assessment Scale (BPFAS; Crist and Napier‐Phillips 2001) a widely used clinical parent‐report measure of mealtime and feeding behaviour in clinical samples. These scales (BAMBI, Aut‐Eat and BPFAS), have a strong emphasis on general clinical feeding problems and are not as relevant to neurotypical samples. Whilst the BAMBI is extensively used to measure food rejection in ASC it contains questions relating to extreme emotional and behavioural reactions to foods and mealtimes, for example, *The child is aggressive during mealtimes* (Lukens and Linscheid 2008) which conflates behavioural problems in ASC with the symptoms of avoidant eating.

There are no scales which explicitly measure different aspects of avoidant eating in both children with ASC and TD children, and the current research aimed to carry out two studies to address this gap in the literature. The aim of the first study was to develop the questionnaire (The Food AVERSE Questionnaire) to measure the different aspects of food avoidance behaviour in children, that is suitable for children with and without ASC. The aim of the second study was to examine whether any subscales of food avoidance differed between children with and without ASC and whether reports of early feeding problems were associated with scores on the developed Food AVERSE Questionnaire.

## Study One: Development of the Food AVERSE Questionnaire

## 2 Introduction

Food avoidance is common in young children in both nonclinical and clinical populations (Cardona Cano et al. 2015; Bourne et al. 2022). Current measures of avoidant eating in children are based on samples of typically developing children and do not represent the spectrum of behaviours seen at mealtimes (e.g., Wardle et al. 2001; Rioux et al. 2017), and/or are focused on general clinical feeding problems (Crist and Napier‐Phillips 2001). The aim of study was to produce a new reliable scale that could be useful in both populations that represents the behaviours seen in avoidant eating.

## 3 Methods

### 3.1 Participants

3.1.1

Participants (*n* = 336) were recruited online from parent groups and organisations, including groups for parents of children with ASC. Of those, 92 had a reported ASC diagnosis and 244 were reported as typically developing. There were 159 girls and 187 boys in the sample aged 5–12 years, with a mean age of 7.23 years (SD 2.31). The majority identified as being white British (*n* = 305, 87%), with the rest of the sample identifying as Black/Black‐British (*n* = 7, 2%), Asian/Asian‐British (*n* = 11, 3%), Arab/Arab British (*n* = 5, 1%), Mixed heritage (*n* = 13, 4%) and seven individuals preferring not to disclose their ethnicity. At the time of initial data collection (2019), ARFID was a clinical diagnostic classification in DSM 5 (American Psychiatric Association 2013), however, there were no diagnostic tools or pathways in the NHS to enable consistent diagnoses. Therefore, children were not recruited or classified on the basis of an ARFID diagnosis.

### 3.2 Materials

#### 3.2.1 Development of the Food AVERSE Questionnaire

The Food AVERSE Questionnaire was developed as a parent‐report measure of avoidant eating behaviour in children. Items were originally devised as part of a pre‐screening process for avoidant eaters in the Feeding Clinic at Birmingham Children's hospital, run by Dr Gillian Harris, consultant clinical psychologist and Sarah Mason, specialist lead speech and language therapist. The Food AVERSE Questionnaire is based on the commonly observed eating problems in clinically referred children. The items were adapted and confirmed through extensive literature searching of the characteristics of avoidant eating across both nonclinical and clinical populations.

The first edition of the Food AVERSE Questionnaire contained 40 items relating to several aspects of food avoidance; food neophobia (Pliner 1994), familiar food refusal, food appearance evaluation, emotional reactions to foods, sensory issues related with food, and ritualistic behaviour at mealtimes.

Early screening of items by an ASC specialist (MP) and a specialist in food rejection in normal populations (HC), removed items that were repetitive or unclear to produce a 36‐item scale. All of the scale's items are phrased as statements, and the response answer is a 5‐point Likert scale ranging from 1 (Never or Strongly Disagree) to 5 (Always or Strongly Agree) depending on the wording of the questions. Question 4 follows a slightly different response wording e.g., ‘Finds messy play (e.g., hand painting/muddy, outdoor play)’ (1) ‘Extremely enjoyable’ to (5) ‘Refuses to participate’. The statements 16: ‘Eats food prepared by anybody’ and 22: ‘Constantly samples new and different foods’ are reverse‐scored.

#### 3.2.2 Child Food Rejection Scale

The CFRS (Rioux et al. 2017) is an 11‐item parental report questionnaire with two subscales of food neophobia (six items) and pickiness (five items). The scale was developed to differentiate between food neophobia and picky eating and has been confirmed as reliable and valid. All items were scored on a five‐point scale (1–5). Scores on the food neophobia subscale range from 6 to 30, and on the pickiness subscale range from 5 to 25. In both subscales, a higher score indicates higher neophobic and picky behaviours.

#### 3.2.3 Food Frequency Questionnaire

The food frequency questionnaire (FFQ, adapted from Cooke et al. 2006) is a short parental report questionnaire which measures typical weekly food consumption of particular food groups. In this study we measured food groups which are known to be liked or disliked by children with high food rejection, these were (1) Vegetables (not including potatoes, (2) Fruit, (3) Sweet snack foods (Cakes, biscuits, sweets, chocolate), (4) Savoury snack foods (crisps, crackers, nuts) (5) Carbohydrates (pasta, rice, potatoes bread products) (6) Protein (meat, fish, beans, meat substitutes) and (7) Dairy (milk, cheese, yoghurts and non‐dairy alternatives). All items are measured with a frequency score in response to the question, *How often does your child eat a portion of the following items in a typical week? A portion is the amount that your child can hold in their hand*. All scores are divided by 7 to generate an average daily consumption score for each food group.

#### 3.2.4 Sensory Sensitivity

The subscales of the Sensory Profile were used to measure taste, smell and texture sensitivity (Dunn 1999). These three subscales comprise 18‐items from the original 60‐item questionnaire which assesses sensory processing among children. These three scales have been identified in previous research by the authors as representing sensory sensitivity in the domain of eating (Coulthard et al. 2016). The scale is high in internal consistency, with a range of 0.64–0.77 (Brown and Dunn 2010). The items are rated on a 5‐point scale, ranging from 1 (almost never) to 5 (almost always). The scores on the items are summed to get a total, and this can range from 16 to 80. A higher score indicates higher taste, smell and tactile sensory sensitivity.

#### 3.2.5 Cognitive Flexibility

The Flexibility Scale‐Revised (FS‐R, Strang et al. 2017) is a parent report scale with 27 items pertaining to dimensions of cognitive flexibility across five subscales; Social Flexibility (5 items), Routines and Rituals (5 items), Transitions/Change (7 items), Special Interests (6 items), and Generativity (4 items). The FS‐R has a four‐point ordinal Likert scale for each item: 0 = no, 1 = somewhat, 2 = very much, 3 = always, with a high score indicating a higher amount of cognitive inflexibility.

### 3.3 Procedure

Approval for this study was obtained by the research ethics board of the University of Birmingham (ERN 13‐0310). Parents read an information letter before giving consent. Parents' participation was voluntary, and the information collected about their child was anonymous. Data was collected through a link to an anonymous survey on Qualtrics.

### 3.4 Data Analysis

Data analysis was conducted using the SPSS 27 (IBM SPSS 2020). To evaluate the Food AVERSE Questionnaire a PCA (Principal Components Analysis) with varimax rotation was conducted. Histograms and Kolmogorov–Smirnov analysis showed that the majority of the data were not normally distributed. Therefore, either non‐parametric tests were conducted or tests were bootstrapped (*n* = 1000 iterations). Spearman Rho correlations were carried out to examine associations between the subscales of the newly developed questionnaire, and the measures of food rejection (pickiness and food neophobia), inflexibility, sensory sensitivity and food frequency scores (vegetable, fruit, protein, dairy, carbohydrate, sweet snack food and savoury snack food consumption).

## 4 Results

### 4.1 Factor Analysis on the Food AVERSE Questionnaire

Exploratory principal components analysis with varimax rotation was performed to reveal the intercorrelation of the initial 36 items. Examination of the scree plot showed that the curve trailed off after three factors, with another drop after four factors before a stable plateau was reached. According to the Kaiser criterion, factors with eigenvalues greater than 1 were retained. From reviewing the alternative factor models and their loadings, five items were removed as they either did not fit the loading criteria or they did not load conceptually onto the factors. These items were; ‘Is a messy eater’, ‘Does not like dry type of foods (e.g., crackers, soft crisps)’, ‘Refuses to eat foods of a specific colour’, ‘Finds messy play (e.g., hand painting/muddy, outdoor play) enjoyable’, ‘Eats food prepared by anybody’. The final questionnaire had 31 items, which were entered into a second PCA factor analysis. This PCA suggested the retention of three factors explaining 58.87% of the variance. The three factors identified with eigenvalues > 1 were: Factor 1‐ Food avoidance, Factor 2‐ Rigid‐inflexible and Factor 3‐ Texture sensitive (Table 1).

**Table 1:** Factor loadings for the three subscales of the Food AVERSE Questionnaire.

		Factor		
	Question text	1	2	3
1	Pulls faces of disgust towards food	**0.654**	0.237	0.199
2	Becomes anxious around new foods	**0.733**	0.371	0.163
3	Vomits/gags in response to foods they dislike	**0.571**	0.387	0.092
4	Spits out foods they dislike	**0.634**	0.290	0.061
5	Completely refuses to eat foods they dislike	**0.772**	0.200	0.168
6	Only eats foods of specific brands or flavours	**0.631**	0.423	0.303
7	Refuses to eat food which looks different than usual (e.g., broken biscuits, bruised fruits)	**0.559**	0.459	0.232
8	Has a diet that consists of only a few foods	**0.678**	0.369	0.327
9	Is unwilling to eat many of the foods that our family eats at mealtimes	**0.783**	0.241	0.291
10	Is fussy or picky about what he/she eats	**0.815**	0.238	0.302
11	*Constantly samples new and different foods	**0.555**	0.024	0.213
12	Does not trust new food	**0.790**	0.247	0.291
13	Won't try a new food if she/he does not know what is in it	**0.756**	0.255	0.223
14	Is afraid to eat things she/he has never had before	**0.793**	0.303	0.197
15	Only eats foods of a specific colour	0.270	**0.520**	0.216
16	Would feel upset/irritable if different foods were touching	0.433	**0.534**	0.129
17	Only eats/drinks from a particular plate/cup/spoon etc.	0.171	**0.795**	0.159
18	Becomes upset/irritable when mealtimes don't follow typical routine	0.251	**0.738**	0.136
19	Refuses food if the packaging is changed	0.351	**0.723**	0.250
20	Feels upset/irritable when a meal is not prepared in the usual way (i.e., different ingredients used/baked not fried)	0.553	**0.543**	0.248
21	Eats different foods on the plate in a particular order	0.238	**0.424**	0.299
22	Only eats particular foods in specific places (e.g., Chips only at McDonalds)	0.415	**0.504**	0.273
23	Only eats when specific people are present	0.099	**0.650**	0.286
24	Dislikes having their teeth cleaned – especially at the sides of the mouth	0.130	**0.535**	0.170
25	Keeps food in the side of their mouth, hesitating to swallow it	0.257	**0.597**	0.041
26	Prefers foods which have certain textures (e.g., Smooth)	0.412	0.357	**0.582**
27	Does not like lumpy foods	0.340	0.412	**0.589**
28	Does not like chewy foods (e.g., meat)	0.381	0.382	**0.377**
29	Does not like mashed type of foods (e.g., mashed vegetables, potato)	0.321	0.112	**0.738**
30	Does not like puree smooth foods (e.g., vegetable/fruit smoothies, yoghurt)	0.118	0.219	**0.762**
31	Does not like wet foods (e.g., pasta, foods with sauces)	0.278	0.277	**0.693**
	Eigenvalue	14.92	1.94	1.39
	Percentage of variance	48.1	6.3	4.5

*Note:* Bold denotes loading onto factor.

The first subscale of the Food AVERSE Questionnaire was food avoidance (48.1%) which consisted of 15 statements (Cronbach's *α* = 0.96) exploring rejection of familiar and unfamiliar foods. The second subscale was rigid‐inflexible (6.3%), which consisted of 10 items (Cronbach's *α* = 0.86) measuring adherence to routines and sameness in mealtime behaviour. The third subscale was texture sensitive (4.5%), which consisted of a set of 6 statements (Cronbach's *α* = 0.86) focusing on food rejection based on texture.

### 4.2 Associations With Food Intake and Sensory/Cognitive Variables

In a subsample of 115 participants, tests of association were carried out between subscales of the Food AVERSE questionnaire, reported child food consumption and sensory/cognitive variables to test convergent validity (see Supporting Information: Appendix 1). This showed that scores on the Food AVERSE Questionnaire were associated with similar measures of high food rejection (picky eating and neophobia), high sensory sensitivity, low flexibility, and lower consumption of fruits, vegetables, proteins, and dairy products. The food avoidance subscale was also associated with a higher consumption of savoury snack foods.

## 5 Discussion

The main aim of this study was to develop a reliable scale to measure food avoidance behaviours in children with and without ASC, ascertain whether there are different factors associated with food avoidance behaviours and examine whether these factors were associated with existing measures related to food rejection. An exploratory factor analysis demonstrated that the Food AVERSE Questionnaire contained three factors which all had good internal consistency (*α* > 0.86). The factors identified map onto slightly different aspects of food rejection eating styles, which were termed food avoidance, rigid‐inflexible and texture sensitive. These factors were all associated with variables that are known to be associated with food rejection, including picky eating, food neophobia, sensory sensitivity and inflexibility (van den Brand et al. 2023). In addition, scores on the subscales were associated with parental reports of eating behaviour consistent with the reported intake often observed in picky eating and food neophobia (Cooke et al. 2006; Yong et al. 2023). In particular, higher scores on the subscales of the Food AVERSE Questionnaire were associated with lower fruit, vegetable, protein and dairy consumption. The food avoidance subscale was associated with higher snack food consumption. It is important to note that this study measured food intake according to parental reports of food groups, rather than food diaries or detailed food frequency questionnaires. Therefore, no conclusions can be drawn as to whether or not the children in the sample were meeting dietary guidelines for nutrient or food group intakes. It is known that many children in the UK may exceed some intakes, such as for protein (Syrad et al. 2016), so lesser intake of protein in avoidant eaters may not be a cause for concern (Gan et al. 2021).

## Study Two: Differences in Early Feeding and the Food Averse Scores in Children With ASC and TD Children

## 6 Introduction

Early feeding stages such as breastfeeding and the introduction to solid foods are very important for the development of eating behaviour in later childhood (Harris and Coulthard 2016) and delays in acquiring feeding skills may indicate a risk for later feeding problems (Ramos et al. 2022; Coulthard et al. 2009). Data from a large prospective data set, the ALSPAC study, found that later picky eating was predicted by initial difficulties in complementary feeding, late introduction to lumpy foods and higher food rejection from 15 months onwards (Emmett et al. 2018). This suggests that avoidant eating may be present in early problems with transitions to solid food, but not with breastfeeding.

There is very little data on the onset of feeding difficulties in children with ASC, and whether early feeding behaviours of children with ASC differ from the early feeding behaviour of their TD peers, but the small body of literature available does suggest that early feeding problems may be apparent. In an epidemiological longitudinal study (Emond et al. 2010) of children later diagnosed with ASC, no significant diet variations were noticed at 6 months old. However, at 15 and 54 months, children who were later diagnosed with ASC were reported as significantly more difficult to feed and were more commonly reported as ‘very picky’ in comparison to TD children. They also increased their selectivity over time, eating fewer fresh fruits, vegetables and salads. No research studies, however, examine whether early feeding problems and transition difficulties are associated with the different factors of avoidant eating within the Food AVERSE Questionnaire. It is believed that sensory sensitivity is moderately inherent (Assary et al. 2024), so it would be expected that avoidant eating with a high sensory component would be evident from a younger age (Coulthard et al. 2016). Conversely rituals and routines around mealtimes and meal presentations may be associated with later problems transitioning onto family foods. As there seems to be some differences between TD and ASC children in relation to when their avoidant eating becomes apparent, it is important to look at whether associations between avoidant eating and early feeding problems differs in these two populations.

The main aim of the second study was to examine whether scores on the Food AVERSE Questionnaire differed between children with and without ASC and whether reports of early problems in feeding were associated with scores on the developed questionnaire. It was hypothesised that children with ASC would present with greater current food avoidant, rigid‐inflexible and texture‐sensitive eating compared to TD children. A second aim of this study was to examine whether reports of early feeding problems in both populations was linked to reports of eating behaviour in later childhood. Based on previous research (Emond et al. 2010), it was hypothesised that children with early feeding problems would have poorer current eating behaviour in both groups.

## 7 Methods

### 7.1 Participants

Parents of 99 children with ASC and 151 typically developing children were recruited through internet‐based parent groups and networks. Parents were asked to state whether their children have received an ASC diagnosis (Autism, Asperger's, Other Autism Spectrum Conditions, Other), or none. Exclusion criteria were those children with medical diagnoses affecting growth, swallowing or feeding. Children who had received an ASC diagnosis but scored ≤ 15 on the Social Communication Questionnaire (*SCQ;* Rutter et al. 2003, *n* = 21) were also excluded as were children from the typically developing group who scored ≥ 15 on the SCQ (*n* = 8). The final sample contained 78 children with ASC (7.88 ± 2.53 [SD] years, 15 [19.2%] females) and 143 typically developing children (6.96 ± 2.43 [SD] years, 72 [50.3%] were female).

### 7.2 Measures

#### 7.2.1 Demographic Information

Parents reported on their children's diagnosis, age, gender, weight, height and ethnicity in addition to their own gender, age, weight, height and education (Table 2).

**Table 2:** Demographic characteristics of the sample of children with ASC (*n* = 78) and TD (*n* = 143).

Variables		ASC (*n* = 78)	TD (*n* = 143)
Child age	Mean(SD)	7.88 (2.53)	6.96 (2.43)*
Child BMI	Mean (SD)	0.99 (1.09)	1.05 (0.92)
Child gender	*n*	15 females, 63 males	72 females, 71 males
Child ethnicity	%	94.9% White British/Other 0% Arab 0% Black/Black British 2.6% Asian/Asian British 2.6% Mixed ethnicity	90.2% White British/Other 2.1% Arab 1.4% Black/Black British 0% Asian/Asian British 4.2% Mixed ethnicity 2.1% Other
Parent age	Mean (SD)	38.77 (6.70)	38.81 (5.81)
Parent BMI	Mean (SD)	27.26 (5.06)	24.83 (4.29)
Parent gender	*n*	73 mothers, 5 fathers	132 mothers, 11 fathers
Parent education level	%	24.4% Post‐Graduate 38.5% University graduate 19.2% A‐Levels 9% GCSEs 5.1% No qualification 3.8% Other	31.5% Post‐Graduate 42% University graduate 9.1% A‐Levels 8.4% GCSEs 6.3% No qualification 2.8% Other

* (Table 2). This was a significant difference (*p* < 0.05).

#### 7.2.2 Social Communication Questionnaire (SCQ; Rutter et al. 2003)

The Social Communication Questionnaire is a 40‐item parent report screening measure that offers a quick, easy, and inexpensive way to routinely screen for autism spectrum conditions (Chesnut et al. 2017). The SCQ was completed by all parents to ensure that children who were in the TD group did not show significant symptoms of ASC and to confirm that those parents reporting ASC had children who reached the criteria for ASC.

#### 7.2.3 Early Eating Behaviour Questions

Parents stated ‘Yes’ or ‘No’ in response to questions asking whether their children presented with feeding problems during each of the following periods: breast/formula feeding, transition to complementary food (weaning), progression from puree to lumpy‐solid foods, progression from lumpy solid food to family food, during the finger food stage and in the toddler period when children were 18 months old (Tables 3, 4, 5).

**Table 3:** Differences in the frequency of reported problems in early feeding stages between children with ASC (*n* = 78) and TD children (*n* = 143).

Early feeding problems	ASC *n* (%)	TD *n* (%)	*Χ* ^2^
Breast\Formula feeding problems	32 (41%)	45 (31.5%)	2.03
Difficulties in the transition to complementary food (weaning)	14 (17.9%)	23 (16.1%)	0.16
Difficult transition from pureed to lumpy food	19 (24.4%)	22 (15.4%)	2.69
Difficult transition from lumpy solids to family food	23 (29.5%)	21 (14.7%)	6.93*
Difficulties with eating during the finger food stage	16 (20.5%)	18 (12.6%)	2.43
Difficulties with eating during the toddler feeding period (around 18 months)	25 (32.1%)	17 (11.9%)	13.33**

Current food avoidance	**ASC mean (SD)**	**TD mean (SD)**	**Bootstrapped CI**
Food avoidance	3.74 (0.98)	2.75 (1.12)	0.71, 1.28**
Rigid‐inflexible	2.70 (0.87)	1.65 (0.59)	0.84, 1.27**
Texture sensitive	3.14 (0.98)	2.16 (0.90)	0.69, 1.22**
Total food avoidance	3.19 (0.82)	2.19 (0.78)	0.77, 1.23**

* *p* < 0.05.

** *p* < 0.001.

**Table 4:** Associations between subscales of the Food AVERSE Questionnaire and early feeding problems in a sample of TD children (*n* = 143).

		Food avoidance	Rigid‐inflexible	Texture sensitive
Breast\Formula feeding problems	B, (Bootstrapped CI)	−0.08 (−0.48, 0.32)	0.13 (−0.09, 1.72)	0.01 (−0.32, 0.38)
Difficult transition to complementary food	B, (Bootstrapped CI)	**0.87 (0.31, 1.46)**	**0.47 (0.15, 0.79)**	**0.94 (0.46, 1.41)**
Difficult transition from pureed to lumpy	B, (Bootstrapped CI)	0.67 (−0.09, 1.34)	0.16 (−0.22, 0.51)	**0.76 (0.13, 1.35)**
Difficult transition from lumpy to family	B, (Bootstrapped CI)	**1.13 (0.25, 1.74)**	**0.74 (0.16, 1.30)**	0.54 (−0.30, 1.11)
Difficulties during the finger food stage	B, (Bootstrapped CI)	−0.22 (−1.05, 0.55)	0.09 (−0.40, 0.55)	−0.39 (−1.25, 0.38)
Difficulties during the toddler feeding period	B, (Bootstrapped CI)	0.57 (−0.11, 1.27)	**0.41 (0.04, 0.78)**	0.48 (−0.09, 1.06)

* *Note:* Bold values denote statistical significance.

**Table 5:** Associations between subscales of the Food AVERSE questionnaire and early feeding problems in a sample of children with ASD (*n* = 78).

		Food avoidance	Rigid‐inflexible	Texture sensitive
Breast\Formula feeding problems	B, (Bootstrapped CI)	−0.02 (−0.44, 0.41)	0.10 (−0.30, 0.48)	0.24 (−0.20, 0.66)
Difficulties in the transition to complementary food	B, (Bootstrapped CI)	0.25 (−0.28, 0.72)	0.15 (−0.38. 0.63)	**0.63 (0.02, 1.10)**
Difficult transition from pureed to lumpy food	B, (Bootstrapped CI)	**0.60 (0.15, 1.02)**	0.20 (−0.43, 0.92)	**0.53 (0.01, 1.06)**
Difficult transition from lumpy to family food	B, (Bootstrapped CI)	**0.52 (0.12, 0.97)**	0.42 (−0.08, 0.98)	**0.73 (0.30, 1.21)**
Difficulties during the finger food stage	B, (Bootstrapped CI)	0.28 (−0.21, 0.73)	**0.66 (0.06, 1.22)**	0.24 (−0.37, 0.74)
Difficulties during the toddler feeding period	B, (Bootstrapped CI)	**0.74 (0.35, 1.16)**	**0.72 (0.32, 1.19)**	0.44 (−0.02, 0.94)

* *Note:* Bold values denote statistical significance.

#### 7.2.4 The Food AVERSE Questionnaire

The Food Averse Questionnaire is described in study 1. There were three main subscales used in the present study, food avoidant, rigid‐inflexible and texture sensitive. All items were scored on a five‐point scale from ‘strongly disagree’ to ‘strongly agree’; a higher score indicating higher avoidant eating behaviours.

### 7.3 Data Analysis

Initial descriptive analyses (Chi‐square, Mann–Whitney U) were conducted to compare and describe the population characteristics of the sample. No age associations with, or sex differences between, scores on the Food AVERSE questionnaire (see appendix for full demographic details) were found. Chi‐square was then used to examine whether there were differences in frequency of early feeding problems at each stage between children with ASC and TD children. Bootstrapped independent *t*‐test analyses were used to examine differences between ASC and TD groups in current eating problems. Multiple hierarchical regression with bootstrapped confidence intervals was carried out to examine which early feeding problems predicted the three subscales of the Food AVERSE Questionnaire (food avoidant, rigid‐inflexible and texture sensitive). Each feeding problem was entered hierarchically as a step in the regression in chronological order (milk feeding, weaning, puree to lumpy transition, lumpy to family food transition, finger food transition, toddler period). The analyses were run for each subscale of the Food AVERSE Questionnaire for the ASC group and the TD group separately.

## 8 Results

### 8.1 Descriptive and Preliminary Analysis

Mann–Whitney U analysis indicated that the mean age of children with ASC was higher than that of the TD children (Table 2). Child age and Food AVERSE scores were not associated therefore age was not controlled for in further analyses. There were more boys (*n* = 134) than girls (*n* = 87) in the overall sample, and there were more boys (*n* = 63) than girls (*n* = 15) in the ASC group (*p* < 0.001). As no statistically significant gender differences were found in the Food Averse scores in the TD group (*p* = 0.06) or the ASC group (*p* = 0.27), gender was not controlled for in further analysis. There were no statistically significant differences in parents' age, gender, educational level or ethnicity between the two groups.

### 8.2 Differences in Early Feeding Behaviour and Current Food Averse Scores Between Children With ASC and TD Children

There were no differences between children in the ASC and TD groups in retrospectively reported problems in the breast/formula feeding period, the transition to complementary food, the transition from puree to lumpy food or during the finger feeding stage. However, parents of children with ASC reported that their children presented significantly more problems than did the children from the TD group during their transition from lumpy solids to family food, and around 18 months (during the toddler feeding period).

Children in the ASC group also presented with significantly more current eating problems, as demonstrated by the total Food AVERSE questionnaire score, than did the children in the TD group. Similarly, children with ASC scored significantly higher in all subscales of the Food AVERSE questionnaire and were reported to present significantly more food avoidant, rigid‐inflexible and texture sensitive eating styles.

### 8.3 Associations Between Early Feeding Problems and Food Averse Scores in ASC Children and Children Who Are TD

#### 8.3.1 TD Children

A hierarchical model was adopted with each variable entered in a different step. B and bootstrapped CI for when the variable is entered into the model. The final model for food avoidance, *F*(6,142) = 4.55 *p* < 0.001, Adj.*R* ^2^ = 0.17, rigid/routine (*F*[6, 142] = 5.77, *p* < 0.001), Adj.*R* ^2^ = 0.20, and texture sensitivity (*F*[6, 142] = 6.36, *p* < 0.001) Adj.*R* ^2^ = 0.22.

In TD children, all three Food AVERSE scores were associated with problems transitioning to complementary feeding (about 6 months). Texture sensitivity was associated with transitioning from puree to lumpy foods. Food avoidance and rigid‐inflexible eating were also associated with the transition to family foods in the second year of life.

#### 8.3.2 ASC Children

Three regressions were carried out to examine whether early feeding problems were associated with scores on the three subscales of the Food AVERSE Questionnaire for ASC children. A hierarchical model was adopted with each variable entered in a different step. B and bootstrapped CI for when the variable is entered into the model. The final model for food avoidance, F(6, 76) = 3.43, *p* < 0.005, Adj.*R* ^2^ = 0.23, rigid‐inflexible (F[6, 76] = 4.00, *p* < 0.002), Adj.*R* ^2^ = 0.26, and texture sensitive (F[6, 70] = 3.86, *p* < 0.002), Adj.*R* ^2^ = 0.25. It was found that texture sensitivity on the Food AVERSE Questionnaire was associated with reports of earlier feeding problems in the transitions to complementary feeding and to lumpy foods. Food avoidance and rigid‐inflexible scores were associated with later feeding problems with transitions to toddler foods and family mealtimes.

## 9 Discussion

The aim of the second study was to examine whether any subscales of food avoidance differed between children with and without ASC and whether reports of early problems in feeding were associated with scores on the developed Food AVERSE Questionnaire. We found support for the hypothesis that children with ASC would present with a more problematic early feeding background than TD children, supporting findings of previous studies (Baraskewich et al. 2021; Emond et al. 2010 Mahmoud et al. 2021). It was found that children with ASC showed more avoidant eating across all three subscales (food avoidance, rigid‐inflexible and texture sensitive) compared with TD children.

Children with ASD were reported to have more early feeding problems than TD children in only two of the six stages; transitioning from lumpy to family foods (around 12 months), and during the toddler feeding period (around 18 months; Emond et al. 2010). There is some research suggesting that feeding problems in infancy could be an indicator of a subsequent diagnosis of autism (Keen 2008; Laud et al. 2009; Putnick et al. 2022); Twachtman‐Reilly et al. 2008). Based on previous literature, it could be expected that children with ASC would present with a higher frequency of early feeding problems, which would become more prevalent closer to the onset of autism symptomatology (12‐18 months old) (Ozonoff et al. 2010). It could be that children with ASC symptomatology may have had significantly greater difficulty in adapting to the feeding expectations of the family and their environment at this age. At this point, the child is expected to learn mealtime behaviours by ‘modelling’ parents' and siblings' eating (Palfreyman et al. 2015; Smith et al. 2020). It could be that the increasing social importance of mealtimes and social cues are less influential for ASC children (Harris 2000), and consequently, avoidant eating behaviours become more apparent in relation to other children of a similar age.

There was a pattern of association between the subscales of the Food AVERSE Questionnaire, and reports of early feeding problems in both groups of children (ASC and TD). Higher scores on the texture sensitive subscale were associated with earlier solid feeding problems during the introduction to solid foods and the transition from puree to lumpy foods. This finding corresponds with research that suggests that tactile sensory sensitivity may affect very early weaning behaviour (Coulthard et al. 2016) and is present early in development as a difference in how the sensory world is perceived and responded to (Ben‐Sasson et al. 2010). In TD and ASC children, food avoidance and rigid‐inflexible eating scores were associated with transitions from lumpy to family foods. It is likely that this transition stage contains a substantial transition in the sensory properties of the foods eaten, as well as the way that many children are fed, leading to anxiety and avoidance (Harris and Coulthard 2016). Future research should examine the nature of these transitions, and whether they can be managed to lessen the onset of later food avoidance behaviours.

## 10 General Discussion

The main aim of this study was to develop a reliable scale to measure food avoidance behaviours in children and ascertain whether there are different factors associated with food aversion. A second aim was to examine whether scores on the Food AVERSE Questionnaire differed between children with and without ASC and whether reports of early problems in feeding were associated with scores on the developed questionnaire.

This is the first measure of food avoidance in children that has developed subscales, rather than having a unitary score, to examine the different factors associated with food avoidance. The scale and its subscales show good internal reliability and convergent validity with existing scales of food rejection (Rioux et al.^2017^; Wardle et al. 2001), sensory sensitivity (Dunn 1999), inflexibility (Strang et al. 2017) as well as intake of food groups (Cooke et al. 2006). A strength of this study was that the scale was based on over 20 years of clinical experience by one of the authors (GH) combined with extensive knowledge of the literature and represents the breadth of avoidant eating behaviours seen in clinical and nonclinical samples.

The subscales were associated with different earlier reports of feeding problems. In particular, problems with texture sensitivity in eating were associated with reports of early feeding difficulties, whereas reports of avoidant and rigid‐inflexible eating were associated with reports of later feeding difficulties. This supports the possibility that there may be different profiles of food avoidance, with sensory sensitivity‐based rejection presenting at an earlier age. There is some emerging research in clinical cases of ARFID, which suggests different profiles of presentation may exist with sensory processing presenting as a distinct early‐onset subtype (Sanchez‐Cerezo et al. 2024). Future research could examine whether different types of food avoidance have different trajectories and may respond to different, tailored strategies.

This study has some limitations. Children in the ASC group were significantly older than the TD group, and although age did not have any significant effect on the Food AVERSE scores of the sample, it is possible that this age difference may have had an impact on recall of early feeding problems. Recollection of early feeding problems, and in particular, problems within each specific stage of infant feeding, are likely to be influenced by several factors. This will include subsequent diagnosis or experience of other problems in the interim period. In addition, because parents self‐selected into the study, there is a possibility that the data may be influenced by a self‐selection bias. Parents who were more concerned about their child's eating problems may have been more willing to participate in this study, thus the findings may not represent the feeding and eating experiences of a random selection of families, either TD or with ASC.

One of the main issues with the present study is a lack of inclusion of children with a diagnosis of ARFID (Bourne et al. 2020). It is likely that some of the children in both groups would have met the criteria for a diagnosis of ARFID, however, at the time of initial data collection diagnosis of ARFID was not being conducted in the NHS, and there were no adequate screening tools available to screen for ARFID (Archibald and Bryant‐Waugh 2023). It will be extremely important to repeat this study with a sample of children who have secured an ARFID diagnosis. It is likely that in some sub‐types of ARFID (particularly those with poor appetite or sensory sensitivity) there will have been early issues with feeding transitions, and this may be a key early indicator of ARFID.

## 11 Conclusion

This is the first study to produce a parental report measure of avoidant/picky/fussy eating with different subscales to measure the construct of food avoidance. The complexities of food avoidance, and the fact that different factors are implicated in its behavioural expression, is an important outcome of this study. Future research must address these factors and whether there are different profiles of food avoidance, rather than viewing food avoidance as a unitary behaviour.

## Author Contributions

All authors have read and approved the final manuscript. M.P. and H.C. performed the research. M.P. and G.H. designed the research study. M.P. and H.C. analysed the data. M.P. and H.C. wrote the paper.

## Conflicts of Interest

The authors declare no conflicts of interest.

## Supporting information

Supporting information.

## Data Availability

The data that support the findings of this study are available on request from the corresponding author. The data are not publicly available due to privacy or ethical restrictions.
