# Supplementary material for: The Development of the Food Averse Questionnaire: A Measure of Food Avoidance in Children With and Without Autistic Spectrum Conditions
Source: Matern Child Nutr. 2025 Apr 22;21(3):e70025. doi: 10.1111/mcn.70025 (PMC12150162; doi:10.1111/mcn.70025)
Supplement: Supplementary file 1 — Supporting information. [file MCN-21-e70025-s001.docx]

**Appendix 1: Associations between the three subscales of the Food Averse Questionnaire, sensory sensitivity, cognitive flexibility, food rejection and food intake (n=115 children)**

|  | Avoidance | Rigid/Routine | Texture sensitivity |
| --- | --- | --- | --- |
| CFRS Picky | 0.80** | 0.59** | 0.57** |
| CFRS Neophobia | 0.79** | 0.59** | 0.64** |
| Sensory sensitivity | 0.48** | 0.71** | 0.53** |
| Flexibility | 0.52** | 0.70** | 0.56** |
| Vegetable consumption | -0.44** | -0.38** | -0.38** |
| Fruit consumption | -0.38** | -0.35** | -0.29* |
| Sweet snack food consumption | 0.16 | -0.08 | 0.02 |
| Savoury snack food consumption | 0.28* | 0.12 | 0.17 |
| Carbohydrate consumption | -0.04 | -0.21* | -0.16 |
| Protein consumption | -0.27* | -0.27* | -0.25* |
| Dairy consumption | -0.19* | -0.32** | -0.26* |

*p<.05, **p<.001
